# Effect of D-allulose on Fructose-Induced Skeletal Muscle Insulin Resistance

**DOI:** 10.1093/geroni/igaf122.3331

**Published:** 2025-12-31

**Authors:** Teruhiko Koike, Kamal Shahriar, Takamasa Tsuzuki, Ryoichi Banno

**Affiliations:** Research Center of Health, Physical Fitness, and Sports, Nagoya University, Nagoya, Aichi, Japan; Nagoya University, Nagoya, Aichi, Japan; Meijo University, Nagoya, Aichi, Japan; Nagoya University, Nagoya, Aichi, Japan

## Abstract

Consumption of fructose-sweetened beverages, particularly in combination with a high-fat diet, is a major contributor to obesity, diabetes, and nonalcoholic fatty liver disease. Insulin resistance is a key underlying mechanism in the development of these conditions. In particular, ectopic fat accumulation in skeletal muscle plays a crucial role in inducing insulin resistance. This study aimed to investigate the effects of the rare sugar D-allulose, one of the candidates for caloric restriction mimetics, on fructose-induced insulin resistance. Wistar rats were randomly assigned to one of three dietary groups: fructose-free control diet (CD), high-fat diet (HF), or high-fat/fructose diet (HFF). After four weeks, an intraperitoneal glucose tolerance test (IPGTT) and a two-step hyperinsulinemic-normoglycemic clamp (HE-clamp) test were performed. Blood samples were collected after six weeks, and triglyceride levels in the liver and skeletal muscle were measured. Additionally, Western blotting was conducted on skeletal muscle samples. The HFF group was then supplemented with either 5% D-allulose or 5% cellulose, and the same tests were repeated. The HFF group exhibited increased blood glucose levels in the IPGTT and greater systemic and skeletal muscle insulin resistance in the HE-clamp compared to the CD and HF groups. Furthermore, blood triglycerides (TG), hepatic TG, and muscle TG levels were significantly elevated. However, D-allulose supplementation improved insulin resistance in the HFF group and reduced blood TG, hepatic TG, and muscle TG levels. D-allulose may improve insulin resistance by reducing triglyceride accumulation in skeletal muscle. Notably, this effect appears to be independent of its anti-obesity properties.

